# The Relative Importance of Genetic Diversity and Phenotypic Plasticity in Determining Invasion Success of a Clonal Weed in the USA and China

**DOI:** 10.3389/fpls.2016.00213

**Published:** 2016-02-24

**Authors:** Yupeng Geng, Rieks D. van Klinken, Alejandro Sosa, Bo Li, Jiakuan Chen, Cheng-Yuan Xu

**Affiliations:** ^1^School of Ecology and Environmental Sciences, Institute of Ecology and Geobotany, Yunnan UniversityKunming, China; ^2^Ministry of Education Key Laboratory for Biodiversity Science and Ecological Engineering, Institute of Biodiversity Science, Fudan UniversityShanghai, China; ^3^CSIRO Ecosystem SciencesBrisbane, QLD, Australia; ^4^Fundación para el Estudio de Especies InvasivasHurlingham, Buenos Aires, Argentina; ^5^School of Medical and Applied Sciences, Central Queensland UniversityBundaberg, QLD, Australia

**Keywords:** *Alternanthera philoxeroides*, common garden experiment, genetic diversity, invasive species, molecular marker, phenotypic plasticity

## Abstract

Phenotypic plasticity has been proposed as an important adaptive strategy for clonal plants in heterogeneous habitats. Increased phenotypic plasticity can be especially beneficial for invasive clonal plants, allowing them to colonize new environments even when genetic diversity is low. However, the relative importance of genetic diversity and phenotypic plasticity for invasion success remains largely unknown. Here, we performed molecular marker analyses and a common garden experiment to investigate the genetic diversity and phenotypic plasticity of the globally important weed *Alternanthera philoxeroides* in response to different water availability (terrestrial vs. aquatic habitats). This species relies predominantly on clonal propagation in introduced ranges. We therefore expected genetic diversity to be restricted in the two sampled introduced ranges (the USA and China) when compared to the native range (Argentina), but that phenotypic plasticity may allow the species' full niche range to nonetheless be exploited. We found clones from China had very low genetic diversity in terms of both marker diversity and quantitative variation when compared with those from the USA and Argentina, probably reflecting different introduction histories. In contrast, similar patterns of phenotypic plasticity were found for clones from all three regions. Furthermore, despite the different levels of genetic diversity, bioclimatic modeling suggested that the full potential bioclimatic distribution had been invaded in both China and USA. Phenotypic plasticity, not genetic diversity, was therefore critical in allowing *A. philoxeroides* to invade diverse habitats across broad geographic areas.

## Introduction

Since Charles Elton published his classic book on biological invasions in 1958, ecologists have been seeking to determine the factors that make a species an aggressive invader (Williamson, 1996; Nentwig, 2007; Van Kleunen et al., 2015). The ability of alien species to cope with new and heterogeneous environments is essential for their successful establishment in areas outside their native ranges. Phenotypic plasticity, where one genotype can express different phenotypes, is frequently proposed as a characteristic that allows invaders to maintain components of fitness (e.g., growth, survival or fertility; Parker et al., 2003; Richards et al., 2006; Geng et al., 2007a) and ultimately overall fitness (Pichancourt and Van Klinken, 2012) in heterogeneous environments. Another hypothesis is local adaptation by post-invasion evolution (Lee, 2002; Maron et al., 2004; Colautti and Lau, 2015). In this scenario, rapid selection of adaptive genotypes, often facilitated by high levels of genetic diversity, can result in local adaption within the invaded range (Sakai et al., 2001; Lavergne and Molofsky, 2007; Xu et al., 2010a; Barrett, 2015). Both phenotypic plasticity and genetic diversity are effective in generating phenotypic variation in natural populations. Notably, these two mechanisms are not exclusive (Moroney et al., 2013; Si et al., 2014) and it is the total adaptive phenotypic variation, either due to phenotypic plasticity or due to genetic diversity, that will affect the realized performance of alien species in heterogeneous environments (Sultan, 1995; Falconer and Mackay, 1996). Indeed, phenotypic plasticity itself can be the target of natural selection and go through rapid evolution during the different phases of biological invasion (Lande, 2015). Many studies have highlighted the effects of local adaptation or phenotypic plasticity on invasiveness of alien species (Bossdorf et al., 2005; Davidson et al., 2011; Dlugosch et al., 2015), but few have examined the two factors simultaneously. As a result, the relative importance of the two adaptive strategies for invasive species remains largely unknown (Barrett, 2015; Bock et al., 2015).

Clonality is also proposed as an important characteristic in invasive alien plants (Pyšek, 1997). Alien plant populations have higher frequencies of clonality than native species and some of the world's most damaging invasive plants are clonal species (Silvertown, 2008). Furthermore, some clonal plants can occupy disturbed and dynamic habitats across broad geographic distributions (Geng et al., 2007a; Ganie et al., 2015). However, for clonal species, many of the physiologically separated individuals are asexual offspring of the same genet and thus share a common genotype (Ellstrand and Roose, 1987; Silvertown, 2008). Theory predicts that clonal plants will only evolve slowly, making local adaptation more difficult to occur (Barton and Charlesworth, 1998; Silvertown, 2008). Phenotypic plasticity is therefore likely to be an important mechanism allowing clonal species to rapidly invade new and diverse environments (Riis et al., 2010; Keser et al., 2014; Roiloa et al., 2014).

*Alternanthera philoxeroides* is native to South America and has become a problematic species in more than 30 countries (Holm et al., 1997). Interwoven stems can form large, dense monocultures, displacing native vegetation, blocking waterways, and causing significant economic impacts to agriculture (Wang and Wang, 1988; Sainty et al., 1998). In the introduced ranges (e.g., Australia, China, and the USA), *A. philoxeroides* rarely produces viable seeds, reproducing mainly through vegetative structures such as roots and broken stems (Julien, 1995; Holm et al., 1997; Dong et al., 2012). Clonal integration among different ramets is proposed as a mechanism that allow *A. philoxeroides* to colonize habitats that are spatially heterogeneous at fine scale (Liu et al., 2008; Wang et al., 2009; Yu et al., 2009; Xu et al., 2010b; Guo and Hu, 2012; You et al., 2014).

In China *A. philoxeroides* is widely distributed but genetically uniform DNA markers suggest genetic diversity is extremely low (Xu et al., 2003; Ye et al., 2003). This is consistent with its dominantly clonal reproduction. Despite this, *A. philoxeroides* occurs in diverse habitats in China, from fully aquatic (e.g., rivers, reservoirs) to terrestrial (e.g., roadside dry lands), and shows prominent phenotypic variation (Pan et al., 2006). Also, phenotypic plasticity, rather than local adaptation, is responsible for the phenotypic variation with relation to different water availabilities (Geng et al., 2007a). An interesting question is whether the species niche of *A. philoxeroides* in China is mainly determined by phenotypic plasticity and is not limited by low levels of genetic diversity. So far it is not known how the levels of genetic and phenotypic diversity observed in China relates to that present in it's native range and other introduced ranges. Direct comparison of the native and introduced clones is needed to determine the relative importance of genetic diversity and phenotypic plasticity during biological invasions.

In this study, we conducted a series of intercontinental comparisons using *A. philoxeroides* clones collected from both native (Argentina) and two introduced ranges (the USA and China). Our major aim was to examine the relative importance of phenotypic plasticity and genetic diversity in determining invasion extent of *A. philoxeroides* in the USA and China. Molecular marker analyses and a common garden experiment were performed to compare the genetic diversity and phenotypic plasticity of *A. philoxeroides* among the three regions. We expected that the genetic diversity in the introduced ranges was lower, and phenotypic plasticity was higher, than in native range. In addition, we used a bioclimatic model fitted against native range distribution data to examine whether the full potential distribution of the species in the introduced ranges were invaded. If genetic diversity had played an important role in determining the niche range of *A. philoxeroides*, we expected that the lower levels of genetic diversity would limit its potential distribution in the introduced ranges.

## Materials and methods

### Study species and sampling

**Figure 1:**
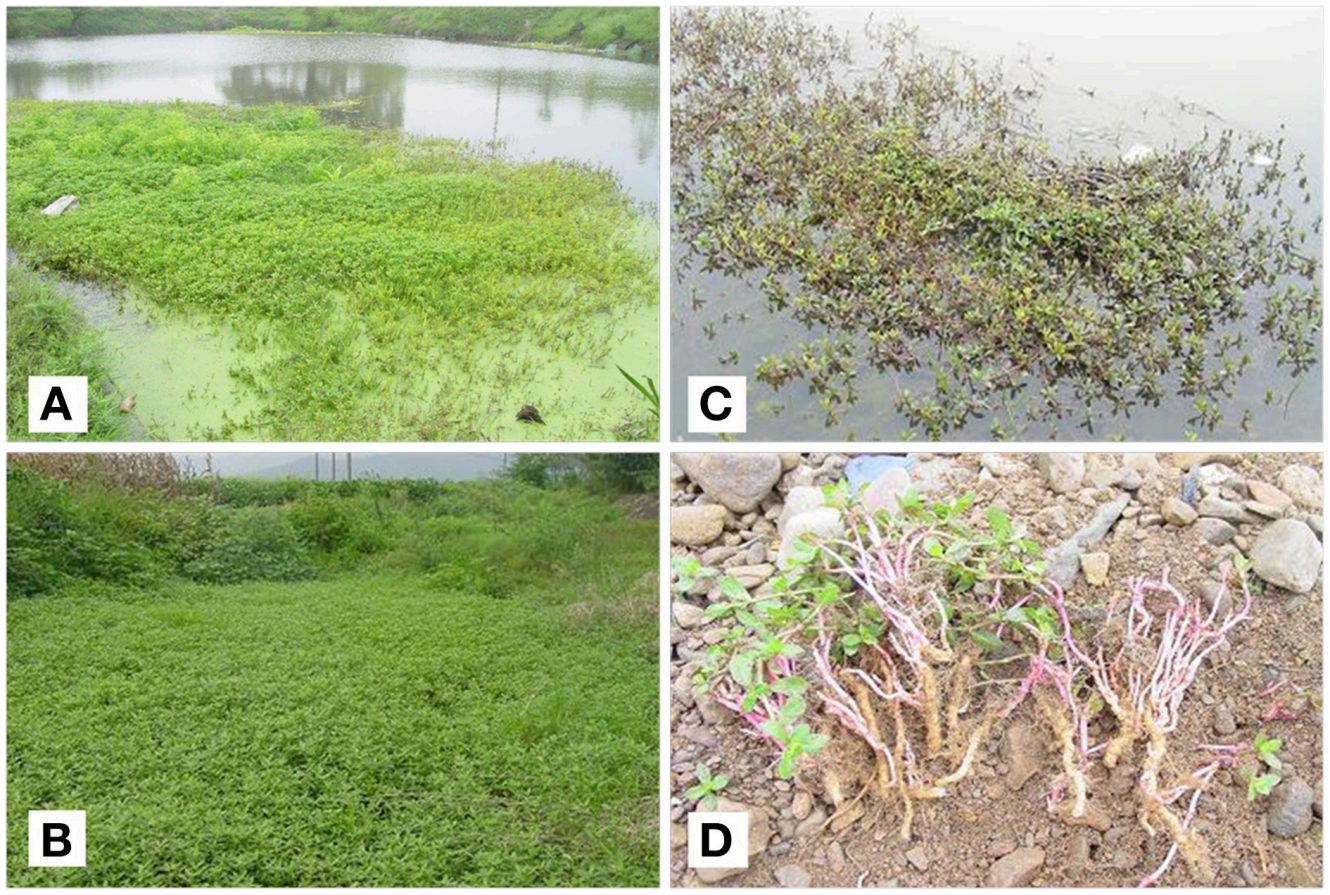
*****Alternanthera philoxeroides*** invades diverse habitats in China and shows different asexual life cycles**. **(A)** Monoculture in aquatic habitat in late summer; **(B)** Monoculture in terrestrial habitat in late summer; **(C)** New shoots grow from underwater stems in aquatic habitat in spring; **(D)** New shoots grow from underground storage roots in terrestrial habitat in spring.

*Alternanthera philoxeroides* (Mart.) Griseb., alligator weed, is a perennial stoloniferous herb. It can thrive in both terrestrial and aquatic habitats (Figure 1). High biomass allocation to root is an important factor determining the performance of *A. philoxeroides* in terrestrial habitats (Wilson et al., 2007; Geng et al., 2007a), including regeneration where cold winters damage most above-ground parts (Figure 1). In contrast, regeneration in aquatic habitats relies mainly on stems (Figure 1).

**Figure 2:**
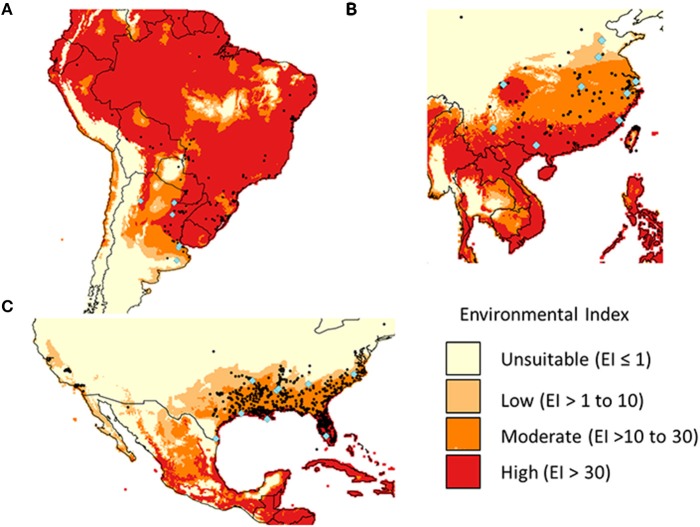
**The potential (Ecolimatic Index) and actual (black points) geographical distribution of ***Alternanthera philoxeroides*** in Argentina (A), China (B), and the USA (C)**. The bioclimatic model was fitted against the native-range distribution data. Sample sites are indicated by blue diamonds.

*A. philoxeroides* is native to South America and has invaded many tropical and subtropical areas across the world (Holm et al., 1997). In Argentina, *A. philoxeroides* is mainly distributed along the Rarana and Uruguay rivers in the north and along the San Borombon and Salado rivers in the center of Buenos Aires province (Sosa et al., 2003; Figure 2). In the USA, *A. philoxeroides* is distributed in several states in the southern coastal plains from Virginia to southern Florida, and westward along coastal areas to Texas and California (Figure 2). In China, *A. philoxeroides* is widely distributed, including in most provinces south of the Yellow River (Figure 2).

A total of 179 *A. philoxeroides* specimens were sampled from its distribution in Argentina (7 sites), the USA (9 sites), and China (9 sites) (Table S1, Figure 2). Specimens at a site were sampled from at least 10 m apart. For each individual, a stem fragment or thickened root was sampled in field. These were grown in a greenhouse in China (Shanghai) for about 6 months before the common garden experiment was performed.

### Molecular marker analysis

To compare the genetic diversity measured by neutral molecular makers, all field-collected samples were analyzed using Inter Simple Sequence Repeat (ISSR) markers, which have proven effective in discriminating different clones of *A. philoxeroides* (Ye et al., 2003). In brief, we extracted total DNA using the cetyltrimethyl ammonium bromide (CTAB) protocol from fresh leaves of *A. philoxeroides* grown in the greenhouse and performed PCR using ISSR primers from the University of British Columbia primer set nine. Eight primers (UBC no. 811, 813, 823, 835, 840, 841, 880, and 887) were selected to genotype *A. philoxeroides.* For each sample, at least two PCR amplifications were performed to evaluate the reproducibility of the bands obtained. Each reaction was carried out in a total volume of 20 μl mixture consisting of 20 ng of template total DNA, 10 mM Tris-HCl (PH 9.0), 50 mM KCl, 0.1% Triton X-100, 2.7 mM primer, 1.5 unit of Taq polymerase and double distilled water. PCR was performed with an Eppendorf Mastercycler programmed for 5 min at 94°C followed by 40 cycles of 45 s at 94°C, 45 s at the appropriate annealing temperature (48–52°C), and 2 min at 72°C. The last cycle was 7 min at 72°C, followed by a 4°C soak. Amplification products were resolved by electrophoresis on 1.5% agarose gels buffered with 1 × TAE.

### Common garden experiment

A common garden experiment (Fudan University, Shanghai; E121°29′-N31°14′) was conducted to compare phenotypic plasticity of *A. philoxeroides* from all sampled ranges in response to different water treatments (aquatic and terrestrial habitat). Each habitat consisted of four rectangular plots (15 × 2 m). The aquatic habitat was simulated using 1 m deep ponds while the terrestrial habitat was simulated with raised garden beds. The aquatic and terrestrial plots were spatially alternated with each other, with adjacent pairs considered as replicates (blocks).

Regional-level phenotypic plasticity was compared by randomly selecting one clone from each sampling site (i.e., 9 from the USA, 9 from China, and 7 from Argentina). As no plants produced seeds in the greenhouse, thick root fragments were used to produce asexual plants as experimental replications. For each of the 25 clones, eight asexual plants with two pairs of leaves grown in pots (30 cm in diameter and 35 cm in depth, containing 1:1 mixture of loam and sand) were allocated randomly to eight plots (i.e., 2 water treatments × 4 replicates) to give a total of 200 pots. Aquatic plants were monitored daily to ensure that the water level remained nearly 2 cm above the pots in ponds. In terrestrial plots, plants received natural precipitation (1200 mm/year) plus supplementary irrigation in continuous sunny days (1L/pot when surface soil in >50% pots are dry).

Plants were harvested after 2 months of growth, which was before any flowers appeared. First, six morphological and physiological traits were measured following the protocol reported in Geng et al. (2007a): (1) leaf length, (2) stem diameter, (3) stem pith cavity diameter, (4) internode length (5) specific leaf area (SLA), and (6) relative chlorophyll content (measured using a chlorophyll meter, Minolta SPAD-502) which gives a value that is well correlated with chlorophyll content. In addition, each individual was separated into four parts: leaves, stems, thick storage roots, and fine roots (i.e. roots with diameter less than 1 mm). All plant materials were oven-dried at 80°C for 48 h and weighed. Then, two allocation traits were obtained, root/shoot ratio and storage root/fine root ratio. The whole experiment was performed in a closed garden equipped with weed mat to prevent plants from escaping into the field.

### Data analyses

#### Analysis of genetic variation in molecular markers and quantitative traits

Genetic diversity was assessed both by neutral molecular markers (then referred to as marker diversity) and quantitative traits under common garden conditions (then referred to as quantitative variation).

In the molecular marker analysis we recorded ISSR bands as present (1) or absent (0) for each sample. Bands of the same molecular weight were considered to represent the same allele at a given locus. This dataset was analyzed in two ways. First, we used Popgene 1.32 (Yeh et al., 1999) to examine the genetic diversity measured by molecular markers at a regional level, using the following genetic variables: the percentage of polymorphic loci (*P*), the Nei's genic diversity index (*He*), and the Shannon diversity index (*I*). We performed a re-sampling procedure to control the confounding effect of uneven sample size (i.e., 21, 32, and 126 for Argentina, the USA and China, respectively). Specifically, we randomly sampled 21 individuals from the USA and China datasets respectively, from which we calculated the regional genetic parameters. This re-sampling procedure was then repeated 30 times, and the average value for each genetic variable based on the sub-dataset was reported along with average based on the whole dataset. Second, PAUP 4.0 (Swofford, 1998) was then used to determine the relationships among *A. philoxeroides* individuals from different geographical origins using neighbor-joining method. Estimates of similarity were calculated using the index of Nei and Li (1979). Bootstrap values for the neighbor-joining tree were calculated using 1000 replicated neighbor-joining searches.

For the quantitative traits in the common garden experiment, we calculated the coefficients of genetic variation (CVg, Houle, 1992) as our estimation of quantitative variation. For each region in each habitat, the coefficient of genetic variation is calculated as CVg = Sqrt (Vg)/M, where Vg was the genetic variance components among clones within a region, and M was the mean value of different clones within a region.

#### Analysis of phenotypic plasticity in quantitative traits

We used the dataset from the common garden experiment that simulated aquatic and terrestrial habitats to compare phenotypic plasticity between the three study regions (Argentina, China and the USA).

First, we examined the reaction norms at regional level by plotting the mean values of all clones from the same continent against two habitat treatments. We performed two-way nested ANOVAs to examine the effects of treatment, region, clone, and treatment-by-region interaction on each univariate trait, in which clone was nested in region as a random factor. The statistical model included the following terms: treatment, region, clone, treatment-by-region, treatment-by-clone, and error term. A significant effect of treatment suggests significant phenotypic plasticity of *A. philoxeroides* in terrestrial vs. aquatic growth conditions while the regional or clonal effect suggests differentiation of *A. philoxeroides* in phenotypic traits among different regions or clones. A significant treatment by region or treatment by clone interaction indicates that the level of phenotypic plasticity is different among regions or clones. We performed *F* tests by testing region effect over clone term; by testing both treatment and treatment-by-region effects over the treatment-by-clone interaction term; and testing treatment-by-clone effects over the error term. We first used Log (initial stem length) as a covariate to examine whether the covariate explained a significant amount of variation. When the covariate was not significant, we performed an ANOVA instead of ANCOVA, and examined the assumptions of homoscedasticity and performed data transformation where necessary.

Second, quantitative analyses of phenotypic plasticity were also performed based on the plasticity index (Schlichting, 1986). Specifically, for each trait of each clone, the plasticity index was calculated as: Ip = (Max (P1, P2) – Min (P1, P2))/Mean (P1, P2), where P1 and P2 were the average values of four replicates of the same clone under aquatic and terrestrial habitats, respectively. We performed one-way ANOVA on these indices to examine whether the effect of region was significant, with clone as error term. When a significant region effect was detected, we conducted *post-hoc* comparisons based on Bonferroni test to examine whether or not the phenotypic plasticity of *A. philoxeroides* in China and USA was significantly different from those from Argentina. As all Chinese clones in common garden experiments proved to be the same multi-locus genotype (i.e., C-Dominant), the plasticity index for Chinese clones might not be independent to each other. To examine the potential effects of pseudoreplication, we also performed a nested ANOVA on plasticity indices, in which region was the main factor and clone was nested in region as a random factor. The overall mean plasticity for Chinese clones was used in this nested ANOVA. In addition we conducted multiple comparisons via *t*-tests. Specifically, the overall mean plasticity index for Chinese clones was used as a fixed value in two single-sample *t*-tests (i.e., USA vs. China mean and Argentina vs. China mean), separately. One two-sample *t*-test was performed when comparing the USA and Argentina. The results of one-way ANOVA, nested-ANOVA, and multiple *t*-tests were similar (see Supplementary Files). For simplification, we reported the result of one-way ANOVA in main text.

Third, to explore the effect of treatment on phenotypic correlation, we examined the Pearson's product correlations between paired traits in aquatic and terrestrial habitats, respectively. For each genotype, trait means were calculated per habitat. Based on the plasticity index, we examined the correlation of plastic responses across habitats among traits (i.e., plasticity integration). The critical probability levels for the correlation coefficients were Bonferroni corrected for multiple comparisons to α/36 = 0.0013 (i.e., there were 36 paired comparisons).

Finally, to provide a multivariate perspective, we examined the overall phenotypic pattern of *A. philoxeroides* from different regions, using principal component analysis (PCA) conducted on the clone mean value of each trait (*n* = 50, 2 treatments × 25 clones). Trait data were standardized prior to PCA. We also performed constrained ordination analysis (e.g., redundancy analysis, RDA), in which two factors variables (i.e., treatment and region) were used as explanatory variables. The result of RDA was highly similar to that of PCA (see Supplementary Files). For simplification, we reported the result of PCA (biplot) in main text. All analyses were carried out with R (R Core Team, 2015).

#### Correlation between genetic and phenotypic dissimilarity

To assess the correlation between molecular markers and quantitative traits, we further examined whether the differences among clones in their quantitative traits were related to their genetic marker dissimilarity. Specifically, we calculated the Euclidean distance matrix based on the quantitative traits of each genotype in terrestrial and aquatic habitats, respectively. The trait data were standardized prior to distance calculations. Mantel tests were then used to assess correlations between the trait matrix and genetic distance matrix based on molecular markers. In addition, the Euclidean distance matrix based on the phenotypic response to treatment (i.e., plasticity) for each genotype across terrestrial and aquatic habitats were also examined using the same method. The distance calculations and Mantel tests were done using the ecodist package (Goslee and Urban, 2007) in R (R Core Team, 2015).

#### Bioclimatic modeling

A bioclimatic model was fitted against the native-range distribution of *A. philoxeroides*. It was then used to test whether the potential distribution in its introduced range (China and the USA) was fully invaded. The model was developed using CLIMEX Version 4.0 (Kriticos et al., 2015) and the world 10 min climate data set downloaded from CliMond (Kriticos et al., 2012). CLIMEX uses temperature and soil moisture data (calculated using rainfall and evaporation). Distribution data was obtained from Global Biodiversity Information Facility (GBIF, global species distribution dataset, http://www.gbif.org/species/3084923), supplemented by China distribution (NSII, China National Specimen Information Infrastructure, http://www.nsii.org.cn/), and the USA distribution (Early Detection and Distribution Mapping System, EDDMapS, http://www.eddmaps.org/distribution/viewmap.cfm?sub=2779). A previously published CLIMEX model for alligator weed (Julien et al., 1995) was modified (Table S2). Temperature parameters were adjusted so that stress only began once conditions were no longer suitable for growth (a CLIMEX requirement not adhered to in the original model), Moisture Index and Temperature Index parameters were tightened as much as possible without affecting the native-range fit, and the cold stress accumulation parameter reduced to better fit the southern-most distribution in Argentina. Outputs (Environmental Index scaled from 0 to 100) from the new model were plotted against distributional data for each region using QGIS 2.12 (Qgis Development Team, 2015).

## Results

### Genetic variation of molecular markers and individual traits

**Figure 3:**
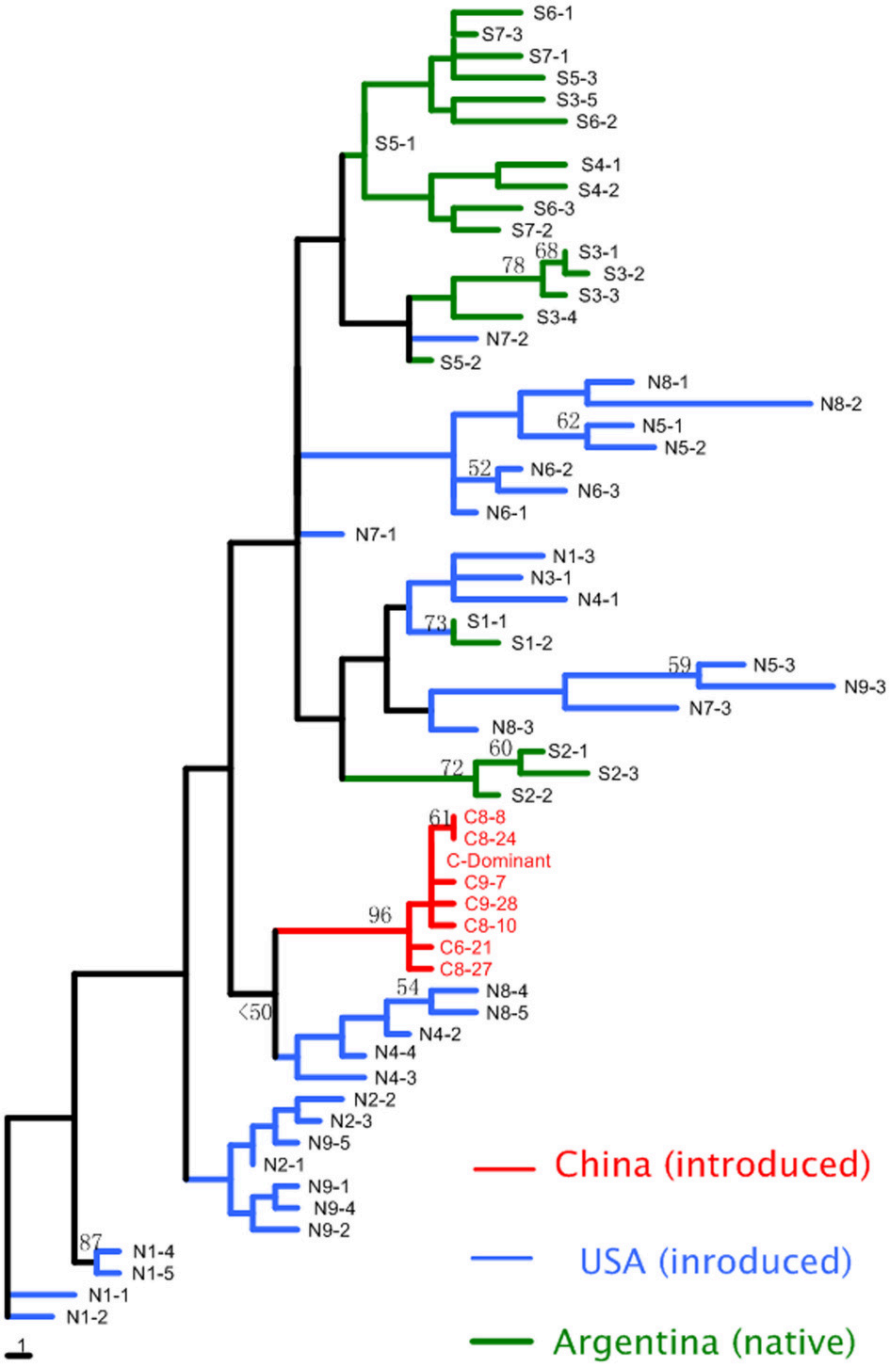
**Neighbor-joining tree of ***Alternanthera philoxeroides*** multi-locus genotypes from Argentina, China, and the USA**. Numbers at nodes represent bootstrap support values (%, only values >50 are shown). All the individuals from China cluster together, with most having the same multi-locus genotype (C-dominance).

A total of 179 *A. philoxeroides* individuals from Argentina (21), the USA (32), and China (126) were analyzed using ISSR markers. The eight ISSR primers generated a total of 60 bands and 61 unique ISSR multi-locus genotypes. For samples from Argentina and the USA, each plant was characterized by a unique multi-locus genotype. In contrast, 94% (119) of the Chinese samples were identical (referred as to “C-Dominant” in Figure 3). The eight Chinese genotypes clustered together as a single well-supported clade in the neighbor-joining tree. This clade was closest to individuals from two sites in the USA (N4 and N8), although bootstrap value was low (Figure 3). In contrast, USA genotypes were represented in many clades, including ones that included Argentine genotypes. Individuals from the same site were usually grouped together, but there was no clear genetic structuring within each region.

All three genetic variables (*P, He*, and *I*) also showed much higher genetic diversity for clones from Argentina and the USA than those from China, even after the confounding effects of different sample size were controlled (Table 1A).

Table 1**Comparison of genetic diversity, measured both by molecular markers (A) and by quantitative traits (B), of ***Alternanthera philoxeroides*** from Argentina, China and the USA**.

**Table:** 

**(A) MARKER DIVERSITY**									
**Population (sample size)**	***P (%)***	***He***	***I***
Argentina (21)	60.00	0.1821	0.2759
USA (32)	69.33^a^ (71.67^b^)	0.2293 (0.2323)	0.3445 (0.3495)
China (126)	2.22 (11.67)	0.0043 (0.0144)	0.0071 (0.0260)

**Table:** 

**(B) QUANTITATIVE VARIATION**									
	**Biomass**	**RSR**	**SFR**	**Leaf-L**	**LIN**	**Stem**	**SPC**	**RCC**	**SLA**
**CV-Terrestrial**									
Argentina	0.1585	0.5030	0.4391	0.2033	0.1036	0.2034	0.4487	0.1268	0.1290
USA	0.1147	0.1981	0.2392	0.1700	0.1575	0.2440	0.4073	0.1268	0.1541
China	0.0763	0.1577	0.2107	0.0807	0.1131	0.0312	0.1968	0.0870	0.0713
**CV-Aquatic**									
Argentina	0.3772	0.2194	0.8221	0.2300	0.1430	0.2335	0.2319	0.1215	0.1613
USA	0.3680	0.2771	0.5291	0.1889	0.1482	0.2116	0.2552	0.1178	0.1787
China	0.2377	0.1254	0.5354	0.0868	0.0930	0.0604	0.0897	0.0668	0.0749

SFR, storage roots/fine roots; RSR, root/shoot; LIN, length of internode; Leaf-L, leaf length; stem, stem diameter; SPC, stem pith cavity; RCC, relative chlorophyll content; SLA, specific leaf area.

*P, percentage of polymorphic loci; He, Nei's genic diversity; I, Shanon's index*.

a Values based on re-sampling dataset with confounding effect of disproportional sample sizes controlled;

b *Values based on original dataset. See details in Methods*.

*CV-terrestrial (-aquatic), coefficients of genetic variation in terrestrial (aquatic) plot*.

Genetic diversity as estimated using quantitative traits measure during the common garden experiment showed patterns consistent with those revealed by neutral molecular markers. For both habitats, quantitative variation was much lower among Chinese clones than among clones from Argentina and the USA for most quantitative traits examined (Table 1B).

### Phenotypic plasticity of individual traits

**Figure 4:**
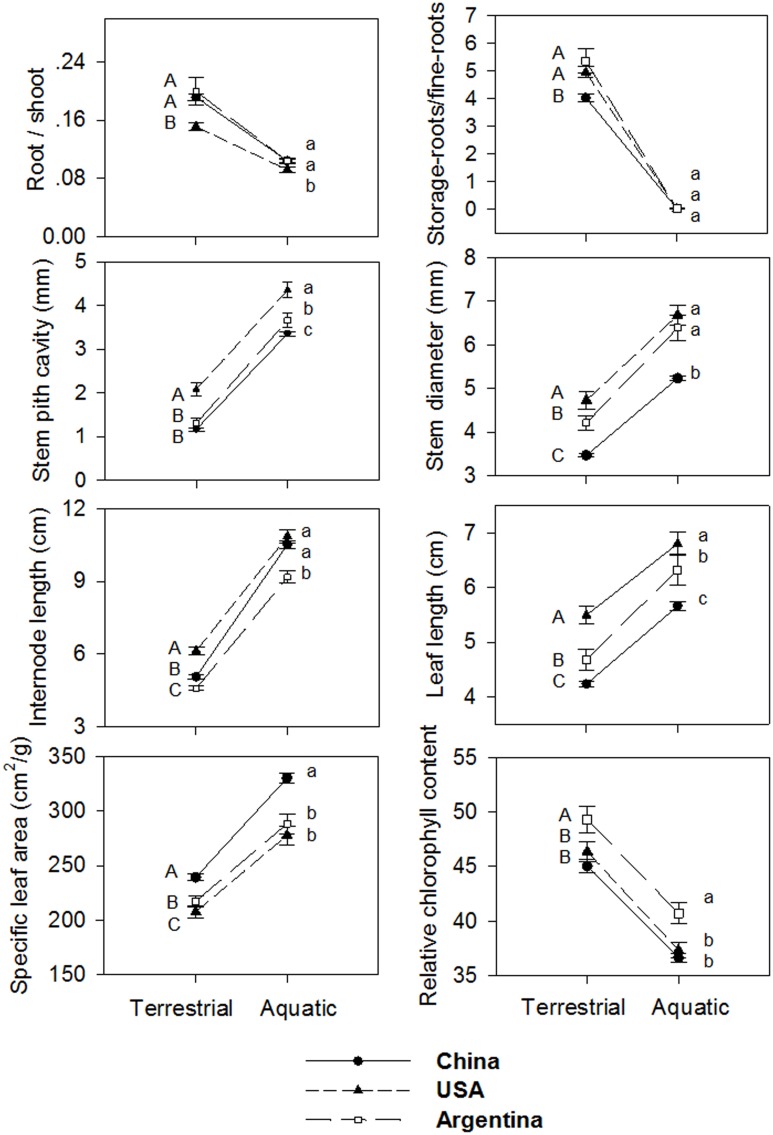
**Reaction norms of ***Alternanthera philoxeroides*** from Argentina, China and the USA against the two habitat treatments tested in the common garden experiment**. Lines are the mean ± 1 SE. Results of *Post-hoc* comparison based on Bonferroni test are shown using capital letters (terrestrial plots) and small letters (aquatic plots). Values sharing the same letter do not differ significantly (α = 0.05). Abbreviations are the same as Table 1.

Generally, plants in aquatic plots had longer leaves, longer internodes, thicker stems and larger stem pith cavity, larger specific leaf area, lower root/shoot ratio, lower relative chlorophyll content, and lower storage root/fine root ratio, than those in the terrestrial plots (Figure 4). The two-way ANOVA revealed significant effects of treatment on all the traits, indicating significant phenotypic plasticity across all regions (Table 2, treatment effect *P* < 0.01), despite significant differences among clones (Table 2, clonal effect *P* < 0.01).

**Table 2:** **Effects of treatment (aquatic and terrestrial), region (Argentina, China and the USA), clone and two-factor interaction on the allocation and morphological traits in ***A. philoxeroides*** in the common garden experiment**.

**Traits**	**Treatment**	**Region**	**Treatment × Region**	**Clone (Region)**	**Treatment × Clone**
	***df* = 1,22**	***df* = 2,22**	***df* = 2,22**	***df* = 22,112**	***df* = 22,112**
***F*-VALUES**					
Ln(biomass)	493.96^**^	0.78	2.84	9.24^**^	2.37^**^
Sqrt(SFR)	5435.36^**^	1.26	2.32	4.13^**^	1.87^*^
Sqrt(RSR)	58.38^**^	5.65^*^	0.54	6.43^**^	9.75^**^
LIN	876.78^**^	13.16^**^	2.60	5.75^**^	2.52^**^
Leaf-L	160.32^**^	4.21^*^	0.32	24.08^**^	2.53^**^
Stem	470.37^**^	4.68^*^	0.96	46.60^**^	2.30^**^
SPC	915.24^**^	4.97^*^	0.79	21.50^**^	1.79^*^
RCC	133.56^**^	2.29	0.09	8.78^**^	2.27^**^
SLA	237.72^**^	6.45^*^	2.25	9.91^**^	2.31^**^

Clone was nested in region as a random factor. Significance levels are given by

* *P < 0.05*,

** *P < 0.01*.

*Abbreviations are the same as Table 1*.

**Figure 5:**
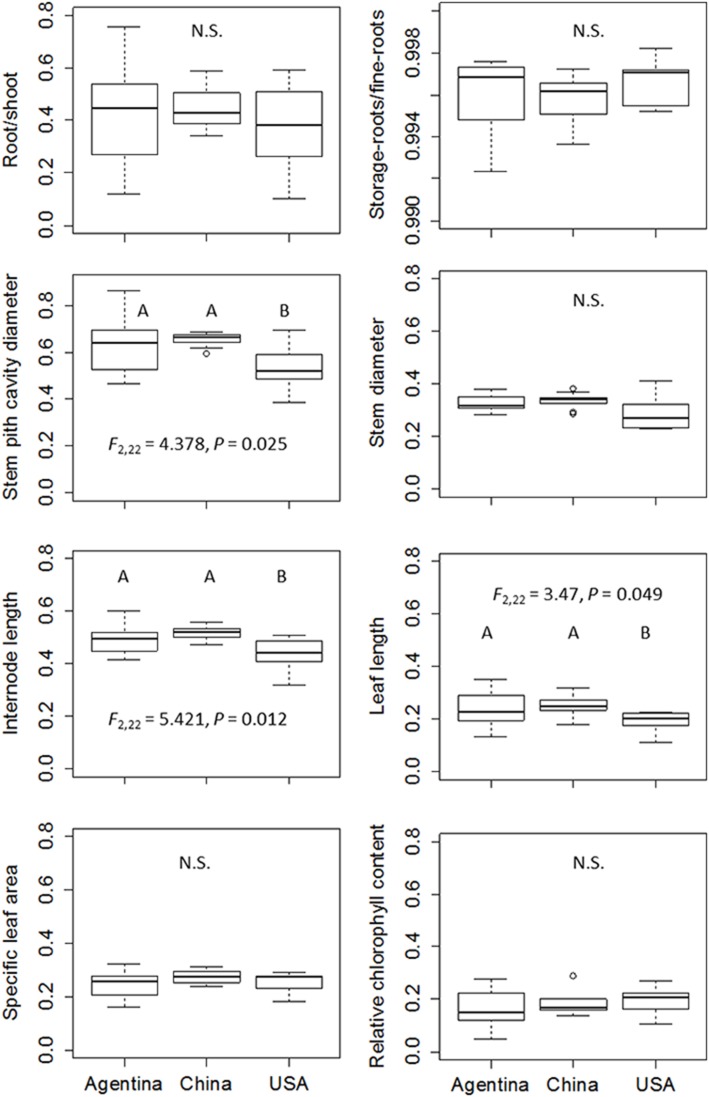
**Comparison of plasticity index of ***Alternanthera philoxeroides*** from Argentina, China and the USA**. Results of *Post-hoc* comparison based on Bonferroni test are shown where significant at α = 0.05. Abbreviations are the same as Table 1.

Contrary to our expectation that clones in introduced ranges had higher phenotypic plasticity than those in the native range, the levels of phenotypic plasticity for examined traits in response to water availability (terrestrial vs. aquatic) were consistent across all three regions (Figure 4). The one-way ANOVA, nested ANOVA, and *t*-tests also found no significant differences among regions for most examined traits when comparing plasticity indices (Figure 5, Table S3). Consistent levels of phenotypic plasticity among regions were further supported by the non-significant effects of treatment by region interaction (Table 2). Although, plants had similar responses at region level, we did detect significant difference in plasticity among clones (Table 2). The clone-level reaction norm suggested that the slope of most traits varied greatly, especially for the clones from Argentina (Figure S1). Indeed, some Argentine clones were more plastic than those from USA and China (Figure 5, Figure S1).

Phenotypic correlation analyses showed that some traits were significantly correlated with each other in both habitats (Figure S2). For example, stem diameter was positively correlated with leaf length and stem-pith-cavity; specific leaf area (SLA) was negatively correlated with leaf length and stem diameter. The treatment changed the phenotypic correlation quantitatively (i.e., increased or decreased the correlation coefficients), but the overall correlation pattern remained unchanged (Figure S2). For the plasticity integration, only one pair of trait plasticity showed a negative correlation (i.e., the stem pith cavity and relative chlorophyll content, Figure S2).

### Correlation between genetic and phenotypic dissimilarity

The Mantel test found the molecular marker distance to be positively correlated with the dissimilarity of quantitative traits in terrestrial habitat (*r* = 0.27, *p* = 0.04) and aquatic habitat (*r* = 0.23, *p* = 0.06, Figure S3). However, we detected no significant correlation between marker distance and dissimilarity of phenotypic plasticity across terrestrial and aquatic habitats (*r* = 0. 15, *p* = 0.29, Figure S3).

### Multivariate (PCA) pattern of quantitative genetic variation and phenotypic plasticity

**Figure 6:**
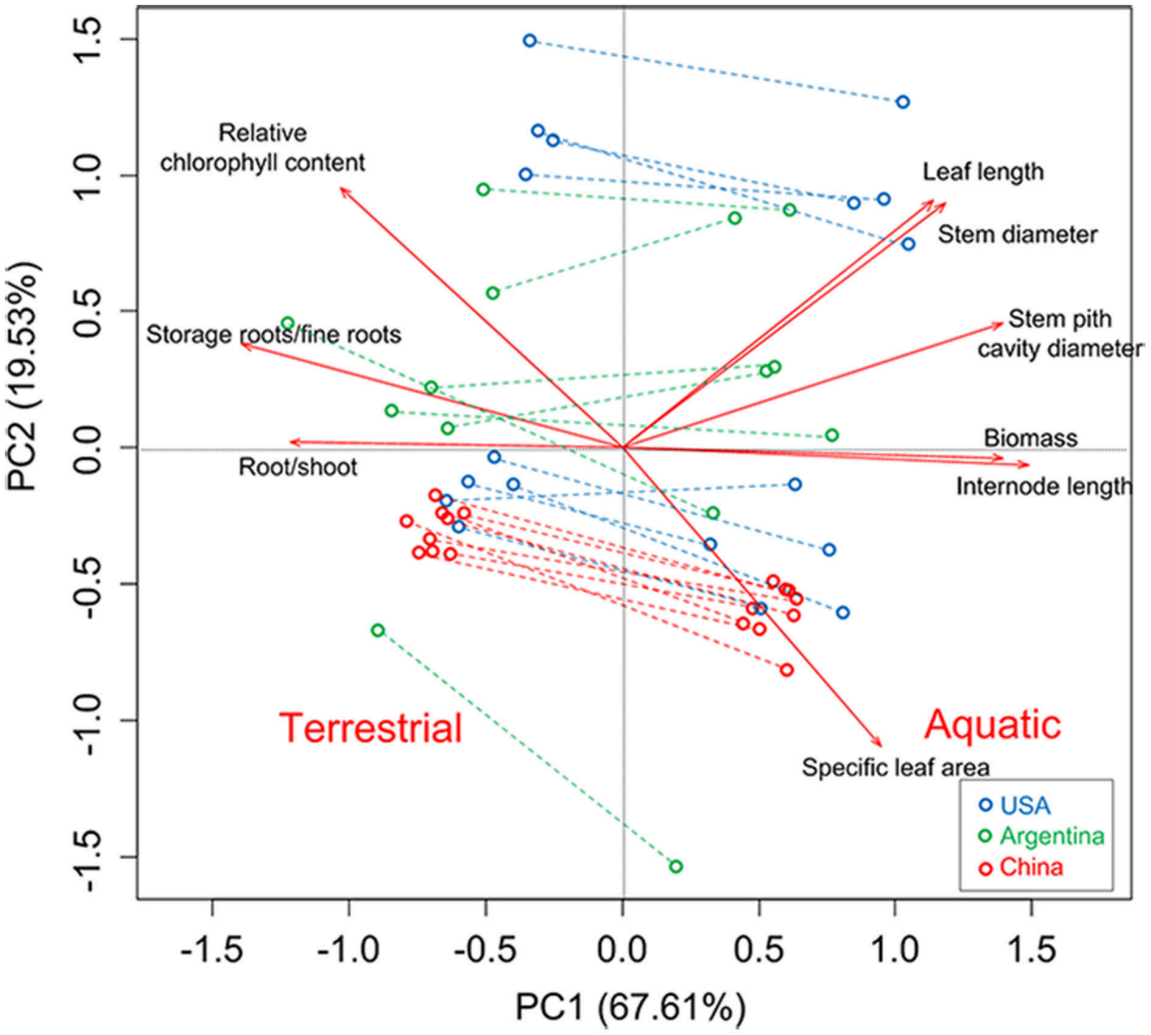
**Biplot for principal components analysis based on quantitative traits**. Each point represents a single clone in a single treatment.

The PCA provided a multivariate perspective and indicated the similar pattern. The phenotypic variation among regions was mainly accounted for by the second principal component, which explained 19.53% of the total variation (Figure 6). Specifically, Chinese clones (red) formed a single cluster; in contrast, USA clones (blue) formed two discrete clusters and Argentina clones (green) were interspersed in the PCA space (Figure 6). The first principal component of PCA clearly separated aquatic and terrestrial treatments in PCA space, indicating that most (67.61%) of the phenotypic variation within the common garden experiment was a plastic response to habitat treatment.

### Bioclimatic modeling

Bioclimatic modeling suggested that the full potential distribution of the species in the introduced ranges were invaded in China and the USA. *A. philoxeroides* occurs in relatively diverse climates within its native range (Argentina), restricted largely by cold stress to the south and west, and heat stress in areas to the north of Argentina (Figure 2). Soil moisture had limited effect in the model as *A. philoxeroides* was present from wet to quite arid climates. The Environmental Index (EI) was high even in areas where *A. philoxeroides* has not been recorded in Argentina. These could not be excluded from the model by further restricting parameters without losing known locations, therefore suggesting non-climatic factors are also important.

The potential distribution in China and the USA was largely restricted by cold stress to the north and heat stress within the range and to the west. In both cases most distribution records occurred within the potential range, although records did extend into areas where cold stress was expected to be too high. There were no extensive areas where EI was moderate to high (above 10) in which *A. philoxeroides* has not yet been reported.

## Discussion

Phenotypic plasticity and genetic diversity have long been proposed to contribute to the invasion success of alien plants, especially clonal invaders, but few studies have tested their relative importance (Barrett, 2015; Bock et al., 2015). In this study, we found high levels of genetic diversity in *A. philoxeroides* in the native range (Argentina) and one introduced range (USA), but not in another introduced range (China). Specifically, genetic diversity in the USA was similar to that in Argentina. In contrast, the levels of genetic diversity in China were extremely low and many individuals collected from geographically distant sites shared the same multi-locus genotype. Contrary to our expectations that clones in introduced ranges had higher phenotypic plasticity than those in native range, the phenotypic plasticity in response to different water availability (terrestrial or aquatic) was similar across all three regions. Despite the different levels of genetic diversity, bioclimatic modeling suggests that the full potential bioclimatic distribution had been invaded in both China and USA. Taken together, our results suggest that the ability of *A. philoxeroides* to successfully invade heterogeneous habitats and broad geographic distributions is the consequence of phenotypic plasticity rather than genetic diversity.

### Comparison of genetic diversity

In this study, we used both molecular markers and quantitative traits to assess the genetic diversity of *A. philoxeroides*. Mantel test suggested that the correlation of the two measures is significant but weak (*r* = 0.27 and 0.23 in terrestrial and aquatic habitat, respectively), which is similar with previous results (*r* = 0.217) based on meta-analysis (Reed and Frankham, 2001). Both molecular markers and quantitative traits revealed a clear pattern that Chinese clones had much lower levels of genetic diversity, in terms of both marker diversity and quantitative variation, than those from Argentina and the USA. The extremely low levels of marker diversity among analyzed Chinese clones are consistent with previously reported results (Xu et al., 2003; Ye et al., 2003; Geng et al., 2007a). The regional-level genetic diversity in USA and Argentina may be underestimated due to smaller sample size, which means the overall pattern of genetic diversity between China and the other two regions may be even more prominent. The levels of genetic diversity of alien species are often shaped by population history (e.g., foundering effect, and whether multiple-introduction had occurred). In this study, all the Chinese samples clustered as a single well-supported clade in the neighbor-joining tree. This suggests Chinese populations may be the result of a single introduction, with the low levels of genetic diversity among Chinese clones being the result of founding effects during invasion. In contrast, the USA clones were scattered on the neighbor-joining tree and were intermingled with Argentina clones, suggesting that the *A. philoxeroides* populations in the USA might have stemmed from multiple introductions. Indeed, the levels of genetic diversity of the clones in the USA were similar to that in Argentina, suggesting no obvious founding effect in the USA.

The level of genetic diversity had little effects on the invasion potential of *A. philoxeroides* to invade its potential distribution within either China (low diversity) or the USA (high diversity) as assessed by a bioclimatic model fitted against the native-range distribution. Especially, the genetic uniformity of Chinese clones did not appear to restrict the geographic and ecological distribution of *A. philoxeroides*. Similar results have also been reported in a few other invasive alien plants in their introduced ranges, e.g., *Pennisetum setaceum* (Poulin et al., 2005), *Rubus alceifolius* (Amsellem et al., 2000), and *Fallopia japonica* (Hollingsworth and Bailey, 2000). Notably, most of these invaders are selfing, or apomixis clonal species, which can usually avoid genetic erosion through Allee effects (e.g., inbreeding depression). Therefore, it seems that the levels of genetic diversity may not be a critical factor limiting the distribution and abundance of clonal invasive plants. So far, several well-documented case studies on post-introduction evolution mainly involved out-crossing or mixed-crossing species, e.g., *Hypericum perforatum* (Hierro et al., 2005), *Phalaris arundinacea* (Lavergne and Molofsky, 2007), and *Sapium sebiferum* (Rogers and Siemann, 2004). Therefore, the role of genetic diversity in invasion success might be variable for plant species with different reproductive modes (e.g., mating system).

### Comparison of phenotypic plasticity

Phenotypic plasticity is frequently envisaged as one of the characteristics that contribute to the adaptability and invasiveness of alien species (Parker et al., 2003; Richards et al., 2006) by allowing them to maintain or increase population growth rates across diverse environments (Pichancourt and Van Klinken, 2012). In this study, we found that all clones, regardless of their geographic origins, showed significant phenotypic plasticity in biomass allocation and morphological traits in response to varying water ability. This may play an important role in shaping its niche breadth in relation to water. In particular, there was no significant correlation between the dissimilarities of genetic markers and plasticity indexes among clones, suggesting the plastic response norm of *A. philoxeroides* in terrestrial vs. aquatic habitats is an inherent (species-level) acclimation to these habitats.

Although, it is not easy to rigorously confirm the adaptive significance of phenotypic plasticity in non-model species (Sultan, 1995), we did find evidence that the phenotypic plasticity of *A. philoxeroides* is not the passive result of growth allometry or resource shortage. First, in a previous study, Geng et al. (2007b) found the plastic root/shoot ratio in response to different water treatments were the result of developmentally active adjustment (i.e., true plasticity) rather than ontogenetic drift along the same developmental trajectory (i.e., apparent plasticity). Second, the phenotypic changes are largely functionally adaptive. For example, terrestrial plants allocated more biomass to roots, and produced smaller and thicker leaves, had shorter internodes, which help plants to better balance the absorption and transpiration of water. Aquatic plants had a much larger stem-pith-cavity, which can act as highly efficient aerenchyma (Voesenek et al., 2006) and also enables the stem mats to float on water (Julien, 1995). Notably, the pattern of trait correlation was qualitatively similar across terrestrial and aquatic habitats (Figure S2). We also detected a significant correlation in phenotypic plasticity between stem pith cavity and relative chlorophyll content. Such phenotypic integration may reflect adaptation within a certain environment (e.g., terrestrial or aquatic habitat) or could be by-products of the genetic/developmental constraints (Pigliucci, 2003), which may constrain the expression and evolution of phenotypic plasticity in dynamic environments (Gianoli and Palacio-Lopez, 2009).

Terrestrial plants allocated much more resources to storage roots than those in aquatic habitats, which may be critical for *A. philoxeroides* to invade into both terrestrial and aquatic habitats. Specifically, *A. philoxeroides* is susceptible to seasonal disturbances (e.g., winter frost) in terrestrial habitats, which often kill all the above-ground parts (Figure 1). Thus, the below-ground storage roots become the indispensable organs that allow plants to resprout and re-produce in terrestrial habitats (Wilson et al., 2007; Geng et al., 2007a). In contrast, regeneration of *A. philoxeroides* in aquatic habitats relies mainly on stems that can often survive winter (Figure 1). Indeed, manipulative experiments suggest that the storage roots had much lower resprout ability in aquatic habitats (Geng et al. unpublished data), suggesting decreased functional importance for storage roots in aquatic habitats. The observed phenotypic plasticity was consistent across the native and two introduced ranges, suggesting it is an inherent (species-level) acclimation pattern for growing in diverse habitats.

Invasive species are expected to be more plastic than their native conspecifics (Parker et al., 2003; Richards et al., 2006), which particularly applies to *A. philoxeroides*, given the extremely low genetic variation and broad niche in Chinese populations. However in this study, we found no evidence of this. Although, plants from different regions had similar plastic responses, we did detect significant difference among clones. Clone-level reaction norms suggested that the slope (i.e., amount of plasticity) varied greatly, especially in the clones from Argentina (native region). Indeed, phenotypic plasticity levels of Chinese and USA clones fell within the variation ranges of Argentine clones. In other words, some native clones were even more plastic than the introduced clones. Previous studies on the comparison of phenotypic plasticity between native and introduced populations/species have produced mixed results. For example, Bossdorf et al. (2005) synthesized 10 case studies, of which half suggested that introduced populations were more plastic than native ones, while the other did not. In more recent meta-analysis studies, (Davidson et al., 2011) found that invasive species were more plastic than non-invasives, while (Palacio-López and Gianoli, 2011) found no significant difference between invasive and native species. Theoretically, it has been proposed that phenotypic plasticity may be favored by natural selection only in the initial phase of invasion, resulting in a transient increase in plasticity; in later invasion phases, plasticity will reduce due to plasticity costs because the novel habitat poses continuous directional selection on the optimum phenotype (Palacio-López and Gianoli, 2011; Lande, 2015). However, such a process of genetic assimilation is less likely to occur in asexual clonal species like *A. philoxeroides.* Indeed, the absence of different plasticity levels between native and introduced populations in *A. philoxeroides* does not seem to be the result of transient and reversible post-invasion evolution, but an inherent characteristic of *A. philoxeroides.*

Phenotypic plasticity may be much more important than genetic diversity in determining the success of clonal invasive species like *A. philoxeroides.* In non-clonal species with high levels of genetic diversity, local adaptation and post-invasion evolution are frequently observed (Lee, 2002; Maron et al., 2004; Novy et al., 2013; Turner et al., 2014). However, in clonal species, the efficiency of natural selection is often constrained and rapid evolution is more difficult to occur (Barton and Charlesworth, 1998; Silvertown, 2008). In the case of *A. philoxeroides*, phenotypic plasticity, rather than genetic diversity, may be critical for the potential to cope with heterogeneous habitats with variable water availabilities and climate conditions, i.e., the basic niche, which can translate into a broader realized niche in introduced ranges when the co-evolved competitors (Gurevitch, 1986) and natural enemies (Louda and Rodman, 1996) are absent. This is partially supported by the bioclimatic model result, which greatly overestimated the native-range distribution of *A. philoxeriodes*, suggesting that other factors such as topography and competition are important in limiting the distribution of *A. philoxeriodes* in the native range. Including other factors in the distribution model, as has been done for similar species (Murray et al., 2012), would therefore help to demonstrate the key factors that lead to the niche expansion of *A. philoxeroides* in introduced ranges.

A biogeographical approach is often proposed to compare the introduced populations with their native counterparts (Bossdorf et al., 2005; Hierro et al., 2005). If we regard biological invasion as a “natural experiment,” the repeated invasion success by some global invaders (e.g., *A. philoxeroides*) provides valuable information akin to experimental replications. In cases where repeated invasions indeed share common features, comparisons among these replications may help identify the relative importance of different factors in determining invasion success. In this study, we compared the genetic diversity and phenotypic plasticity of *A. philoxeroides* across three regions. Our results revealed that the pattern of “lower genetic diversity” in one introduced range (i.e., China) was not found in another introduced range (i.e., the USA), reflecting the heterogeneous nature of biological invasions even for the same invader. In contrast, high levels of phenotypic plasticity were found across all three regions, highlighting the importance of phenotypic plasticity as a common feature underlying successful invasions of *A. philoxeroides*. Accordingly, this multi-region comparative approach, including two or more biogeographical replicates, may be especially indicative for understanding the relative importance of different factors underlying successful invasion.

## Author contributions

YG, BL, JC, and CX designed the research. YG performed the wet lab work. RV performed the climate niche modeling. YG and CX performed the data analysis. YG, AS, CX participated in the sampling. YG, RV, BL, JC, and CX drafted and revised the manuscript. All authors carefully read and approved the final manuscript.

## Funding

This study was supported by the National Natural Science Foundation of China (31000112, 31260055), and the International Foundation for Science (Grant A/4424-1).

### Conflict of interest statement

The authors declare that the research was conducted in the absence of any commercial or financial relationships that could be construed as a potential conflict of interest.
