# Supplementary material for: The Relative Importance of Genetic Diversity and Phenotypic Plasticity in Determining Invasion Success of a Clonal Weed in the USA and China
Source: Front Plant Sci. 2016 Feb 24;7:213. doi: 10.3389/fpls.2016.00213 (PMC4764702; doi:10.3389/fpls.2016.00213)
Supplement: Supplementary file 1 [file Table1.DOCX]

Table S1. Collection sites of native (Argentina) and introduced (China and the USA) ***Alternanthera philoxeroides*** populations.

| Clone ID | Site | | Longitude | | Latitude | Sample size | |
| --- | --- | --- | --- | --- | --- | --- | --- |
| USA：9 sites, 32 clones | | | | | | | |
| N1 | Texas | | | ca. W97°38' | ca. N26°12' | | 5 |
| N2 | Texas | | | ca. W93°57' | ca. N29°54' | | 3 |
| N3 | Florida | | | ca. W81°30' | ca. N26°35' | | 1 |
| N4 | Mississippi | | | W88°47' | N33°16' | | 4 |
| N5 | Mississippi | | | W88°28' | N33°31' | | 3 |
| N6 | Arkansas | | | W92°18' | N34°42' | | 3 |
| N7 | Georgia | | | ca. W84°06' | ca. N34°12' | | 3 |
| N8 | North Carolina | | | W76°26'-78°1' | N35°23'-35°53' | | 5 |
| N9 | Louisiana | | | W90°8' | N29°6 | | 5 |
| Argentina：7 sites, 21 clones | | | | | | | |
| S1 | Chaco | | | W59°29' | S27°07' | | 2 |
| S2 | Santa Fe | | | W59°49' | S29°16' | | 3 |
| S3 | Southern Buenos Aires (SBA) | | | W59°03' | S37°11' | | 5 |
| S4 | Misiones | | | W59°31' | S27°15' | | 2 |
| S5 | Buenos Aires | | | W58°37' | S34°36' | | 3 |
| S6 | Eastern Buenos Aires (EBA) | | | W58°44' | S35°00' | | 3 |
| S7 | Tucuman | | | W65°18' | S26°54' | | 3 |
| China：9 sites, 126 clones | | | | | | | |
| C1 | | JiNan | | E117°00' | N 36°40' | | 6 |
| C2 | | WeiShan | | E116°34' | N 34°27' | | 6 |
| C3 | | ShangHai | | E121°29' | N 31°14' | | 6 |
| C4 | | ChengDu | | E104°03' | N 30°50' | | 6 |
| C5 | | WuHan | | E114°19' | N 30°33' | | 6 |
| C6 | | ZhuJi | | E120°20' | N 29°40' | | 30 |
| C7 | | FuZhou | | E119°18' | N 26°05' | | 6 |
| C8 | | KunMing | | E102°43' | N 25°02' | | 30 |
| C9 | | NanNing | | E108°22' | N 22°48' | | 30 |
