# Supplementary material for: The Relative Importance of Genetic Diversity and Phenotypic Plasticity in Determining Invasion Success of a Clonal Weed in the USA and China
Source: Front Plant Sci. 2016 Feb 24;7:213. doi: 10.3389/fpls.2016.00213 (PMC4764702; doi:10.3389/fpls.2016.00213)
Supplement: Supplementary file 2 [file Table2.DOCX]

**Table S2**. Parameters used in the CLIMEX model for *Alternanthera philoxeroides*. Explanations are provided where values were changed from those published by Julien et al. (1995).

| Parameters | Explanation | | Julien *et al*. (1995) | Our model | Notes |
| --- | --- | --- | --- | --- | --- |
| **Moisture** |  | |  |  |  |
| SM0 | Lower soil moisture threshold | | 0.0 | | Unchanged |
| SM1 | Lower optimal soil moisture | | 0.01 | 0.15 | Altered to tighten fit |
| SM2 | Upper optimal soil moisture | | 5.0 | 1.5 | Altered to tighten fit |
| SM3 | Upper Soil moisture threshold | | 10.0 | 2.0 | Altered to tighten fit |
| **Temperature** |  | |  |  |  |
| DV0 | Lower temp. threshold | | 12.0^o^C | | Unchanged |
| DV1 | Lower optimal temp. threshold | | 25.0^o^C | | Unchanged |
| DV2 | Upper optimal temp. threshold | | 32.0^o^C | 30^o^C | Reduced to remove overlap with TTHS |
| DV3 | Upper threshold temp. | | 36.0^o^C | 33^o^C | Reduced to remove overlap with TTHS |
| **Stress Indices** | |  |  |  |  |
| TTCS | Thresholds of cold stress | | 10.0^o^C | | Unchanged |
| THCS | Rate of accumulation of cold stress | | -0.00035 | -0.00025 | Reduced to increase southern range |
| DTHS | Threshold of heat stress (day-degree) | | Not used | |  |
| DHHS | Rate of accumulation of heat stress (temp) | | Not used | |  |
| TTHS | Threshold of accumulation of heat stress (temp) | | 33.0oC | | Unchanged |
| THHS | Rate of accumulation of heat stress. | | 0.0045 | | Unchanged |

Reference:

Julien, M.H., Skarratt, B., and Maywald, G.F. (1995). Potential geographical distribution of alligator weed and its biological control by Agasicles hygrophila. Journal of Aquatic Plant Management 33, 55-60.
