# Supplementary material for: The Relative Importance of Genetic Diversity and Phenotypic Plasticity in Determining Invasion Success of a Clonal Weed in the USA and China
Source: Front Plant Sci. 2016 Feb 24;7:213. doi: 10.3389/fpls.2016.00213 (PMC4764702; doi:10.3389/fpls.2016.00213)
Supplement: Supplementary file 3 [file Table3.DOCX]

**Table S3.** Comparison of plasticity index among regions. A) One-way ANOVA: region is the main factor. B) Nested ANOVA: region is the main factor and clone is nested into region. C) multiple t-test: two one-sample test (i.e. USA VS China mean and Argentina VS China mean) and one two-sample t-test (i.e. USA VS Argentina). Star indicates significant differences (* P < 0.05; ** P < 0.01).

1. One-way ANOVA

| Plasticity of Traits | Region |  |
| --- | --- | --- |
|  | df = 2,22 | |
| *F* values | | |
| Root/shoot | 0.46 | |
| Storage/Fine roots | 1.00 | |
| Internode | 5.42* | |
| Leaf length | 3.47* | |
| Stem diameter | 2.58 | |
| SPC | 4.38* | |
| RCC | 0.45 | |
| SLA | 1.05 | |

1. Nested ANOVA

| Plasticity of Traits |  | Region |  | Clone (Region) |  |
| --- | --- | --- | --- | --- | --- |
|  |  | df = 2,15 |  | df = 15,7 |  |
| *F* values | | | | | |
| Root/shoot |  | 0.13 |  | 5.24* |  |
| Storage/Fine roots |  | 0.00 |  | 2.00 |  |
| Internode |  | 2.62 |  | 4.77* |  |
| Leaf length |  | 2.56 |  | 1.87 |  |
| Stem diameter |  | 1.28 |  | 2.41 |  |
| SPC |  | 2.00 |  | 12.47** |  |
| RCC |  | 0.88 |  | 9.03** |  |
| SLA |  | 0.17 |  | 3.29 |  |

1. Multiple t-test

| Plasticity of Traits | USA v.s. China |  | Argentina v.s. China |  | USA v.s. Argentina |
| --- | --- | --- | --- | --- | --- |
|  | df = 8 |  | df = 6 |  | df=14 |
| *t* values | | | | | |
| Root/shoot | 1.25 |  | 0.35 |  | 0.45 |
| Storage/Fine roots | 1.84 |  | 0.05 |  | 0.84 |
| Internode | 3.74** |  | 1.05 |  | 1.76 |
| Leaf length | 4.81** |  | 0.40 |  | 1.72 |
| Stem diameter | 2.21 |  | 0.67 |  | 1.44 |
| SPC | 3.92** |  | 0.42 |  | 1.75 |
| RCC | 0.43 |  | 0.68 |  | 0.84 |
| SLA | 1.83 |  | 1.25 |  | 0.29 |
