# Supplementary figures and images for: The Relative Importance of Genetic Diversity and Phenotypic Plasticity in Determining Invasion Success of a Clonal Weed in the USA and China
Source: Front Plant Sci. 2016 Feb 24;7:213. doi: 10.3389/fpls.2016.00213 (PMC4764702; doi:10.3389/fpls.2016.00213)

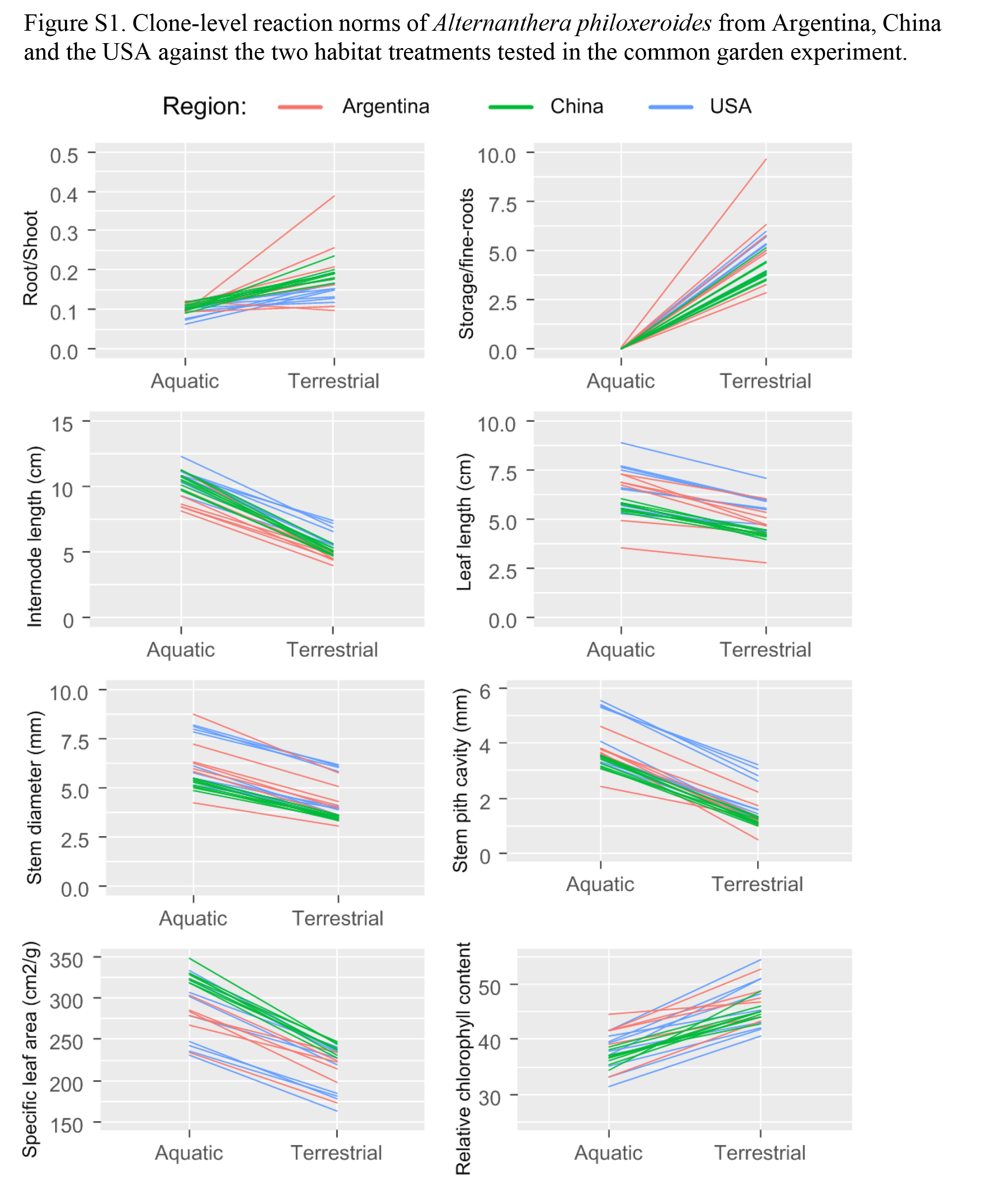

Supplement: Supplementary file 4 [file Image1.JPEG]

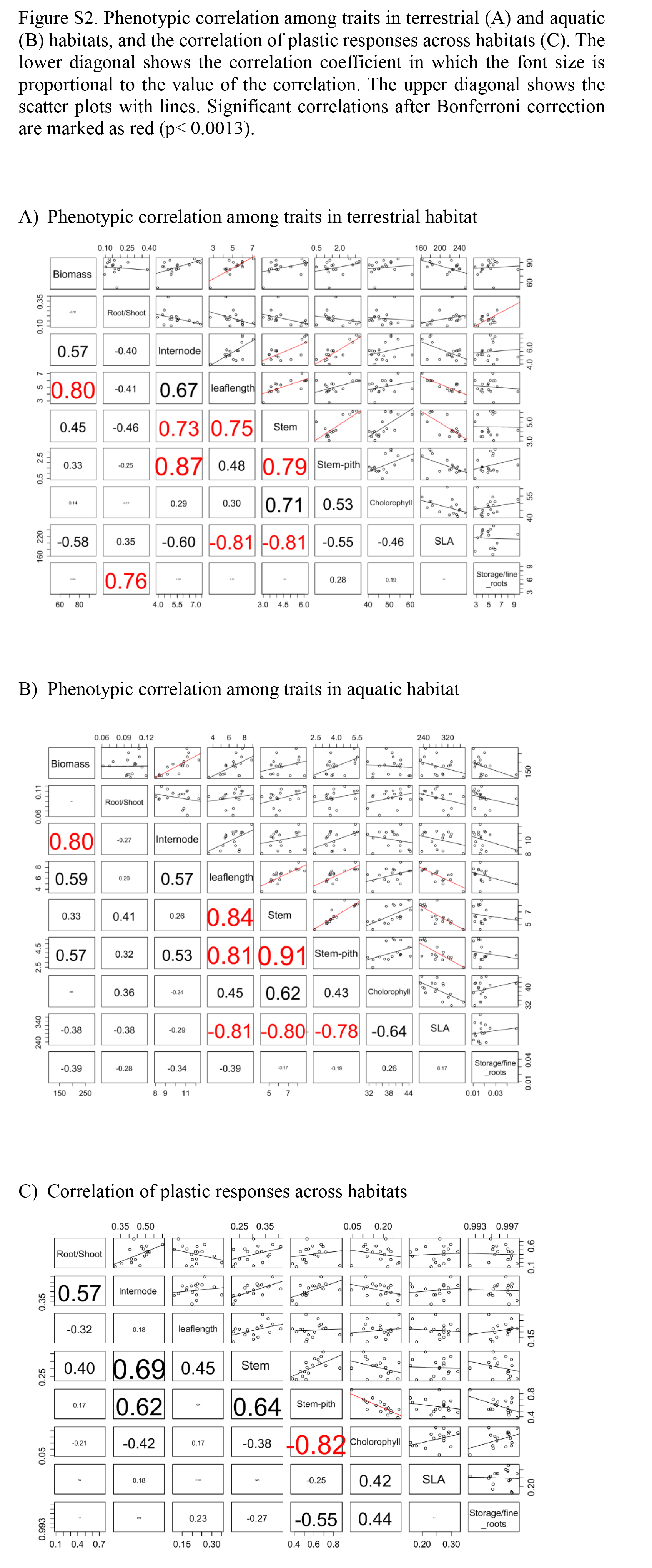

Supplement: Supplementary file 5 [file Image2.JPEG]

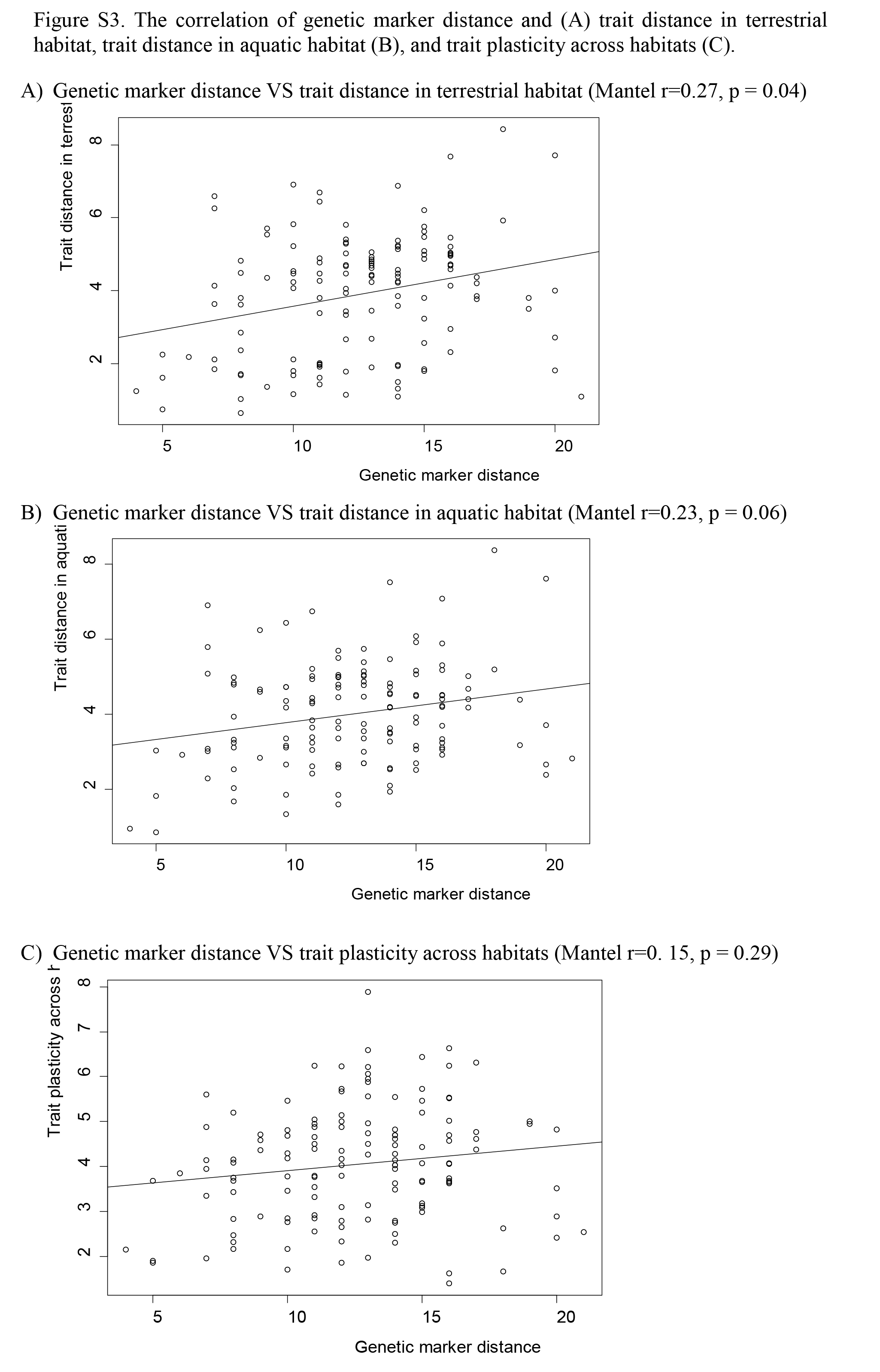

Supplement: Supplementary file 6 [file Image3.JPEG]

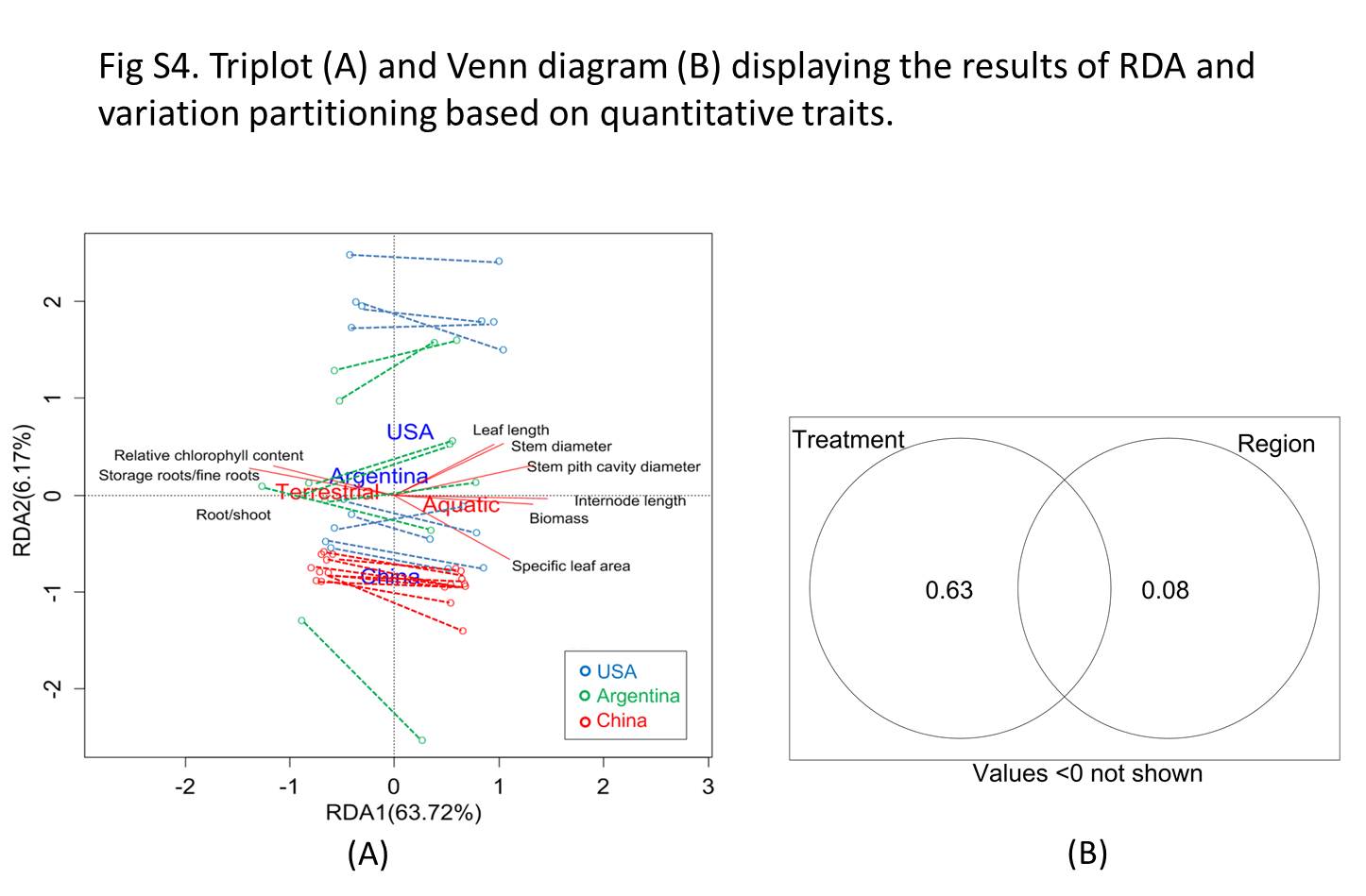

Supplement: Supplementary file 7 [file Image4.JPEG]
